# Association Study of Gene LPP in Women with Polycystic Ovary Syndrome

**DOI:** 10.1371/journal.pone.0046370

**Published:** 2012-10-03

**Authors:** Bo Zhang, Han Zhao, Tao Li, Xuan Gao, Qin Gao, Rong Tang, Jiangtao Zhang, Zi-Jiang Chen

**Affiliations:** 1 Center for Reproductive Medicine, Provincial Hospital Affiliated to Shandong University, Jinan, China; 2 National Research Center for Assisted Reproductive Technology and Reproductive Genetics, Jinan, China; 3 The Key Laboratory for Reproductive Endocrinology of Ministry of Education, Jinan, China; 4 Shandong Provincial Key Laboratory of Reproductive Medicine, Jinan, People’s Republic of China; Baylor College of Medicine, United States of America

## Abstract

**Background:**

Previous genome-wide association study (GWAS) of polycystic ovary syndrome (PCOS) in Han Chinese population has found that SNPs in *LPP* gene were nominally significant in PCOS patients (P around 10E-05). Replication of the GWAS was applied to further confirm the relationship between *LPP* gene and PCOS.

**Methods:**

Three polymorphisms of *LPP* gene (rs715790, rs4449306, rs6782041) were selected and replicated in additional 1132 PCOS cases and 1142 controls. Genotyping of *LPP* gene was carried out by Taqman-MGB method.

**Results:**

In rs715790, the allele frequency is significantly different between the PCOS group and the control group. Meta-analysis showed that the allele frequencies of the three SNPs rs715790 (P_meta_ = 1.89E-05, OR = 1.23), rs4449306 (P_meta_ = 3.0E-04, OR = 1.10), rs6782041 (P_meta_ = 2.0E-04, OR = 1.09), were significantly different between PCOS cases and controls.

**Conclusions:**

Our results suggest that *LPP* gene might be a novel candidate for PCOS.

## Introduction

Polycystic ovary syndrome (PCOS) is the most common endocrine-metabolic disorder affecting 6–8% reproductive-aged women [1]. It is a heterogeneous disease characterized by oligo-ovulation and/or anovulation, clinical and/or biochemical hyperandrogenism and polycystic ovaries on ultrasound [2]. Women with PCOS have a high risk suffering from metabolic syndrome [3], type 2 diabetes (T2D) and cardiovascular diseases [4], [5]. Insulin resistance, present in perhaps 70% of women with PCOS [6], [7], may play an important role in the long-term complications of PCOS. Previously we conducted a genome-wide association study (GWAS) on PCOS in Han Chinese, including single nucleotide polymorphisms (SNPs) with P value less than 10E-06 were replicated, in which three susceptibility loci were confirmed [8]. However, other loci with P value around 10E-05 may also pose potential risks to PCOS and need replication study to confirm the association.

In our GWAS data [8], a pile of SNPs with P value from 10E-04 to 10E-05 were found within gene Lim domain containing preferred translocation partner in lipoma (*LPP*) on chromosome 3q28 (Table S1). The *LPP* gene contains 10 exons and spans a genomic region of more than 400 kb. Studies suggested that LPP was a substrate of the protein-tyrosine-phosphatase 1 B (PTP1B) [9], which is a negative regulator of insulin signaling pathway and plays important roles in the pathogenesis of insulin resistance [10]. Insulin resistance is one of the most important metabolic disorders in women with PCOS. Combining our GWAS data, further replication study is needed to confirm the association of *LPP* gene and PCOS.

To determine the relationship of *LPP* and PCOS, three SNPs rs715790 (T/C), rs4449306 (C/A) and rs6782041(C/T) in *LPP* were genotyped in an additionally independent-sample set of 1132 PCOS cases and 1142 controls. Meta-analysis was performed to combine our GWAS data and the replication data.

**Table 1:** Age and BMI of replicated PCOS cases and Control subjects.

	PCOS	CTRL	P value
**N**	1132	1142	
**Age(years)**	28.54±3.74	31.71±4.77	<0.001
**BMI(kg/m2)**	25.11±4.18	22.77±3.25	<0.001

BMI: body mass index.

## Materials and Methods

### Subjects

The 1132 PCOS cases and 1142 controls were of Han Chinese population, recruiting from the Center for Reproductive Medicine, Shandong Provincial Hospital Affiliated to Shandong University from June 2009 to May 2011.

Recruitment of PCOS was based on the revised 2003 Rotterdam diagnostic criteria, meeting at least two of the following features: chronic oligo-ovulation and/or anovulation; clinical or biochemical hyperandrogenism; and polycystic ovaries on ultrasound. Patients with other diseases such as congenital adrenal hyperplasia, androgen-secreting tumor and Cushing syndrome were excluded. The controls were healthy women, with regular menstrual cycle, excluding hyperandrogenism and polycystic ovaries morphology. Written informed consent was obtained from all subjects. The study was approved by the Institutional Review Board for Reproductive Medicine of Shandong University.

**Table 2:** Allele frequencies in PCOS cases and controls.

SNPs	Allele	Stage	Case	Ctrl	OR	P	OR_meta_	P_meta_
**rs715790**	**T**/C	GWAS	0.433	0.363	1.337	6.97E-05	1.230	1.89E-05
		Replication	0.407	0.374	1.151	0.021		
**rs4449306**	**C**/A	GWAS	0.440	0.382	1.270	9.60E-04	1.100	3.0E-04
		Replication	0.426	0.398	1.122	0.057		
**rs6782041**	**C**/T	GWAS	0.472	0.402	1.324	9.13E-05	1.090	2.0E-04
		Replication	0.455	0.432	1-098	0.114		

Risk allele is shown in bold type.

GWAS: Genome-Wide Association Study.

OR: odds ratio.

The GWAS data and Replication data were combined.

Meta-analysis was performed to analyze the combined data.

OR_meta_: odds ratio by meta-analysis.

P_meta_: P value by meta-analysis.

### Measures

The level of serum testosterone (T) of all subjects were measured by a chemiluminescent analyzer (Beckman Access Health Company, Chaska, MN, USA). 75 g oral glucose tolerance test (OGTT) was carried out for PCOS patients (AU640 automatic biochemistry analyzer; Olympus Company, Hamburg, Germany). The glucose levels and insulin levels at 0 min and 120 min were evaluated. Insulin resistance was estimated by the homeostasis model assessment (HOMA-IR) method according to the formula: fasting glucose (mmol/L) * fasting insulin (mIU/L)/22.5.

**Table 3:** Genotype frequencies in PCOS cases and controls.

SNP	Genotype	PCOS	Control	? 2	P_add_	P_dom_	P_rec_
**rs715790**	**TT**/**T**C/CC	176/576/386	162/522/446	7.534	**0.023**	**0.006**	0.450
**rs4449306**	**CC**/**C**A/AA	194/577/361	185/540/417	5.426	0.066	**0.02**	0.549
**rs6782041**	**CC**/**C**T/TT	218/602/321	217572/376	4.860	0.088	**0.03**	0.769

Risk allele is shown in bold type.

P_add_: P value of additive model (three genotypes).

P_dom_: P value of dominant model [(homozygotes of risk allele + heterozygotes) vs. homozygotes of non-risk allele].

P_rec_ : P value of recessive model [homozygotes of risk allele vs.(heterozygotes+ homozygotes of non-risk allele)].

### SNP Selection

SNPs of *LPP* selected for replication were in accordance with the following criteria: SNPs that exist in the Affymetrix 6.0 chip, can stand for a block; minor allele frequency (MAF) >5% in the Han Chinese population; in the linkage disequilibrium test, SNPs with r^2^<0.8 were selected. All selected SNPs were statistically different (P<10E-04) in our previous GWA study (Table S1) [11]. SNPs rs715790 (T/C, P_GWAS_ = 6.97E-05), rs4449306 (C/A, P_GWAS_ = 9.6E-04) and rs6782041 (C/T, P_GWAS_ = 9.13E-05) in different blocks of *LPP* gene were selected for replication study (Fig. S1).

**Table 4:** Characteristics comparison in PCOS cases using dominant model of rs715790.

characteristics	Risk allele group (N = 719)	Non risk - allele group(N = 364)	t	P	P_adjusted_
**BMI (kg/m^2^)**	25.19±4.21	24.92±4.12	1.016	0.310	
**T (ng/dl)**	52.24±21.73	50.51±21.95	1.238	0.216	0.246
**Glu0′** (mmol/**L)**	5.30±1.00	5.21±0.70	1.601	0.110	0.141
**GLU120′** (mmol/**L)**	6.67±4.63	6.38±1.85	1.129	0.259	0.326
**INS0′** (mIU/L)	11.82±6.87	11.66±8.11	0.325	0.745	0.849
**INS120′** (mIU/L)	60.16±46.42	61.53±47.05	0.452	0.651	0.288
**HOMA-IR**	2.84±1.89	2.86±2.73	0.150	0.881	0.562

Risk allele group is TT plus TC, and the non-risk allele group is CC.

Characteristics were presented by mean±Std.

P_adjusted_ is calculated by logistic regression analysis taking BMI as covariant.

BMI: body mass index; T: testosterone; GLU: glucose; INS: insulin; HOMA-IR: homeostasis model assessment-insulin resistance.

### Genotyping

DNA was extracted from EDTA anticoagulated blood by a QIAamp DNA mini kit (QIAGEN, Hiden, Germany). Three SNPs were analyzed by TaqMan-MGB probe assay (Invitrogen trading, Shanghai) (Table S2). Taqman-MGB fluorescence quantitative PCR was performed on the Light Cycle system (Roche480). Reaction conditions were carried out by initial denaturation at 95°C for 10 min, followed by 45 cycles of denaturation at 95°C for 15 s, annealing at 58°C for 30 s, extension at 72°C for 30 s.

### Statistical Analysis

Clinical characteristics of cases and controls were expressed as means±SD. To evaluate the relationship between each SNPs, pairwise linkage-disequilibrium (LD) (D′ and correlation coefficients r^2^) were calculated by Haploview.

Chi-square test was performed to compare allele frequencies of rs715790, rs4449306 and rs6782041. Combing our previous GWAS data and the present data, meta-analysis was performed using Review Manager 5.1 software, with both fixed and random effects models. Data was presented as odds ratio (OR) and 95% confidence interval (95%CI).

Genotypes of each SNPs were analyzed by additive (+/+ vs. +/− vs. −/−), dominant (+/+ plus +/− vs. −/−) and recessive (+/+ vs. +/− plus −/−). Genotype-phenotype correlation of PCOS was analyzed by independent sample T test.

In phenotype analysis, Chi-square test, independent T test were analysed, and logistic regression analysis used for age and BMI adjustment by SPSS16.0 software (SPSS Inc., Chicago, IL, USA). Statistic significant level was defined as P<0.05.

## Results

Clinical characteristics of PCOS cases and controls are summarized in Table 1. The PCOS group was younger than the control group (P<0.001). And PCOS group had higher body mass index (P<0.001) than control group. Thus, age and BMI were adjusted in the subsequent analysis.

Analyzed by Haploview, Hardy-Weinberg equilibrium tested allele frequencies of the three SNPs were in accordance both in PCOS cases and controls. There were little linkage between rs715790 and rs4449306 (D′ = 0.142, r^2^ = 0.018), rs715790 and rs6782041 (D′ = 0.372, r^2^ = 0.114), rs4449306 and rs6782041 (D′ = 0.668, r^2^ = 0.397). The allele frequencies of rs715790, rs4449306 and rs6782041 were presented in Table 2. In the PCOS group, allele frequency of rs715790 was significantly higher than the control group (P = 0.021, OR = 1.151, 95%CI = 1.021–1.297), even adjusted by age and BMI using logistic regression test (P = 0.024). However, statistical difference of allele frequency was not found in rs4449306 (P = 0.057, OR = 1.122, 95%CI = 0.997–1.262), and rs6782041 (P = 0.114, OR = 1.098, 95%CI = 0.978–1.234). Furthermore, combining previous GWAS data to the present data by meta-analysis (Table 2), significant differences were found in all three SNPs, rs715790 (P_meta_ = 1.89E-05, OR = 1.23, 95%CI = 1.12–1.34), rs4449306 (P_meta_ = 3.0E-04, OR = 1.18, 95%CI = 1.08–1.29), and rs6782041 (P_meta_ = 2.0E-04, OR = 1.19, 95%CI = 1.09–1.30).

Genotype of the three SNPs were analyzed by chi-square test (Table 3). In the additive model, significant difference was discovered only in rs715790 (P = 0.023). In dominant model, significant difference was found in all three SNPs, rs715790 (P = 0.006), rs4449306 (P = 0.02) and rs6782041 (P = 0.03). However, there was no significant difference in recessive model. Of all three models, the dominant model was most effective for genotype analysis.

The dominant model of genotype was thus used to evaluate the clinical characteristics in PCOS patients. In rs715790 (Table 4), there was no statistical differences for T levels between risk allele group and non-risk allele group. After adjusted by BMI impact, the glucose levels and insulin levels showed no significant differences between risk allele group and non-risk allele group. There were no differences in HOMA-IR between the two groups.

## Discussion

In our previous GWA study [8], several loci with P value less than 10E-06 have been identified; However, other loci with P value around 10E-05 are also worthy of investigation, just as *YAP1* gene, which we previously confirmed as another susceptibility gene for PCOS [11]. In this study, we performed a replication study of SNPs in *LPP* gene, and confirmed the plausibility that *LPP* could be a new candidate gene for PCOS.

Three SNPs were carefully selected and one of them, rs715790, was identified to be significantly associated with PCOS. In GWAS data, rs715790 (P_GWAS_ = 6.97E-05) was significantly different between PCOS and controls. Meta-analysis of previous GWA study and the replication data still showed significant difference (P_meta_ = 1.89E-05) in allele frequency. The other selected SNPs rs4449306 and rs6782041 were not replicated, but remain statistically different in meta-analysis study.

*LPP* encodes Lim domain proteins subfamily that are characterized by an N-terminal proline rich region and three C-terminal Lim domains. LPP, as a substrate of PTP1B, may participates in insulin signaling pathway through binding to PTP1B. Binding of insulin to its receptor evokes autophosphorylation of the receptor on tyrosines in the kinase regulatory domain, activating the insulin receptor tyrosine kinase, which phosphorylates the various insulin receptor substrate proteins that trigger the downstream of insulin signaling events [12]. In the insulin signaling pathway, acting as a negative regulator, PTP1B could dephosphorylate the activated insulin receptor [10]. Studies showed that, in obesity, PTP1B expression was increased, which might worsen insulin resistance in those people [13]. PCOS cases have more serious insulin resistance than age-matched controls but independent of BMI [14], [15]. Thus, whether and how LPP functions in this pathway still needs further and extensive studies. Here, *LPP* was confirmed to be a plausible candidate for PCOS, however, no association was found between characteristic insulin resistance and *LPP* gene. The possible reason is that our enrolled subjects were of reproductive age (the average age is 28.54), and at that time few of them suffered from insulin resistance or type 2 diabetes, and this may cause type II error.

Overall, this study indicates that *LPP* is a novel candidate for PCOS. Nevertheless, further studies are warranted to replicate the association patterns in larger cohorts with different genetic background. Functional studies should be considered to explore more meaningful insights on the role of *LPP* gene towards PCOS.

## Supporting Information

Figure S1 **LD plots for SNPs in LPP gene.** P_GWAS_ represent the P-values of GWAS. Values in the box show the squared correlation coefficient (r^2^) between the SNPs. Significant SNPs and haplotype blocks are shown in red (P<0.05). Data were from HapMap database (CHB; http://snp.cshl.org/).
(TIF)

Table S1 **SNPs in GWA study of LPP.** SNPs for replication are shown in bold type. Ctrl: Control; OR: odds ratio.
(DOCX)

Table S2 **Probes and primers of the three SNPs.** F: forward; R: reverse.
(DOCX)
