# Supplementary figures and images for: Association Study of Gene LPP in Women with Polycystic Ovary Syndrome
Source: PLoS One. 2012 Oct 3;7(10):e46370. doi: 10.1371/journal.pone.0046370 (PMC3463595; doi:10.1371/journal.pone.0046370)

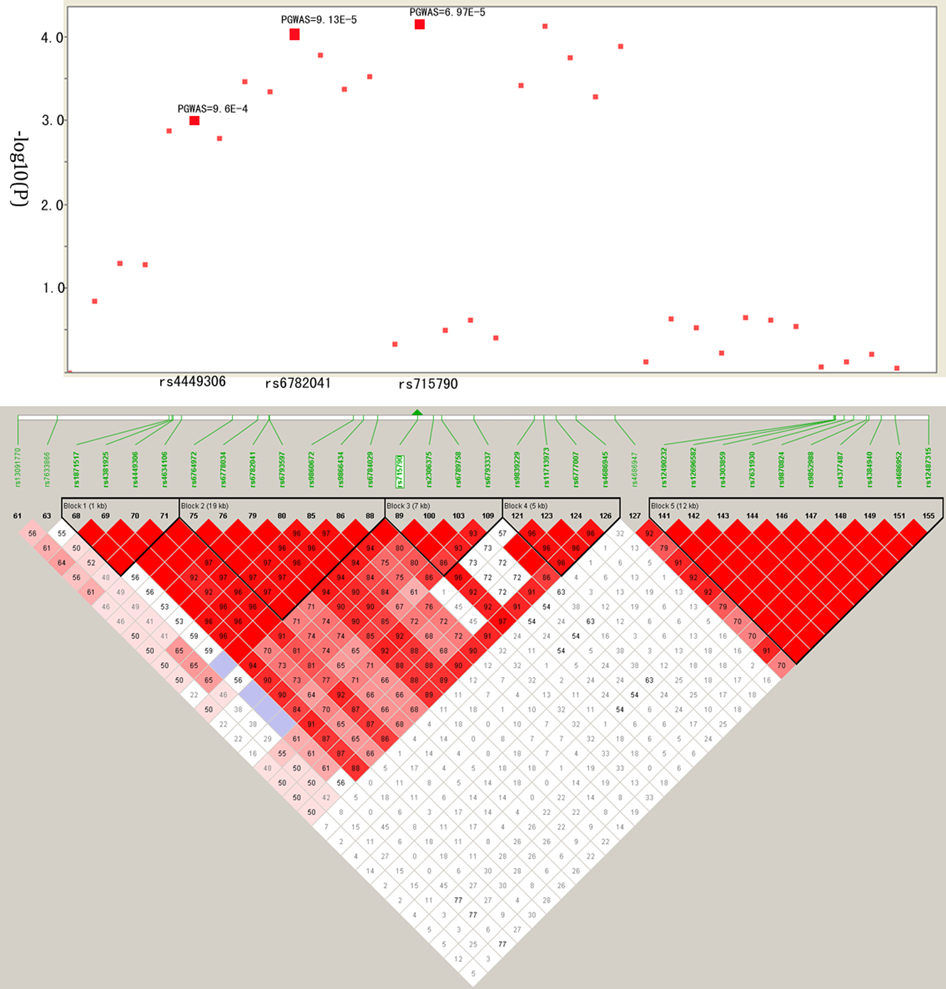

Supplement: Figure S1 — LD plots for SNPs in LPP gene. PGWAS represent the P-values of GWAS. Values in the box show the squared correlation coefficient (r2) between the SNPs. Significant SNPs and haplotype blocks are shown in red (P<0.05). Data were from HapMap database (CHB; http://snp.cshl.org/). (TIF) [file pone.0046370.s001.tif]
