# Supplementary material for: Association Study of Gene LPP in Women with Polycystic Ovary Syndrome
Source: PLoS One. 2012 Oct 3;7(10):e46370. doi: 10.1371/journal.pone.0046370 (PMC3463595; doi:10.1371/journal.pone.0046370)
Supplement: Table S1 — SNPs in GWA study of LPP. SNPs for replication are shown in bold type. Ctrl: Control; OR: odds ratio. (DOCX) [file pone.0046370.s002.docx]

**Table S1. SNPs in GWA study of LPP**

| LPP | SNP | Alleles | PCOS | Ctrl | Chi-square | P | OR |
| --- | --- | --- | --- | --- | --- | --- | --- |
|  | rs13091770 | T/G | 0.2993 | 0.2752 | 2.214 | 0.1367 | 1.125 |
|  | rs7633866 | T/C | 0.3714 | 0.3379 | 3.905 | 0.04814 | 1.158 |
|  | rs1871517 | A/C | 0.1066 | 0.08596 | 3.839 | 0.05008 | 1.269 |
|  | rs4381925 | C/T | 0.4744 | 0.4176 | 10.36 | 0.001286 | 1.259 |
|  | **rs4449306** | **C/**A | **0.4399** | **0.3822** | **10.9** | **9.60E-04** | **1.27** |
|  | rs4634106 | G/C | 0.4681 | 0.4123 | 10.02 | 0.001548 | 1.255 |
|  | rs6764972 | C/T | 0.4439 | 0.3812 | 12.88 | 3.33E-04 | 1.296 |
|  | rs6778034 | G/A | 0.444 | 0.3826 | 12.36 | 4.38E-04 | 1.289 |
|  | **rs6782041** | **C/**T | **0.4715** | **0.4025** | **15.31** | **9.13E-05** | **1.324** |
|  | rs6793597 | G/A | 0.471 | 0.4045 | 14.28 | 1.58E-04 | 1.31 |
|  | rs9860672 | C/A | 0.4 | 0.3396 | 12.51 | 4.04E-04 | 1.297 |
|  | rs9866434 | T/A | 0.3976 | 0.3356 | 13.17 | 2.84E-04 | 1.307 |
|  | rs6784029 | T/C | 0.1093 | 0.101 | 0.5758 | 0.4479 | 1.092 |
|  | **rs715790** | **T/**C | **0.4328** | **0.3634** | **15.82** | **6.97E-05** | **1.337** |
|  | rs2306375 | T/C | 0.1077 | 0.09668 | 1.059 | 0.3034 | 1.128 |
|  | rs6789758 | G/A | 0.1077 | 0.09489 | 1.448 | 0.2288 | 1.152 |
|  | rs6793337 | T/C | 0.1141 | 0.1043 | 0.7774 | 0.3779 | 1.106 |
|  | rs9839229 | C/T | 0.3228 | 0.2653 | 12.73 | 3.61E-04 | 1.32 |
|  | rs7614199 | T/A | 0.4296 | 0.3608 | 15.76 | 7.18E-05 | 1.334 |
|  | rs11713973 | C/T | 0.32 | 0.2597 | 14.12 | 1.71E-04 | 1.342 |
|  | rs6777007 | G/A | 0.3184 | 0.2625 | 12.13 | 4.97E-04 | 1.312 |
|  | rs4686945 | G/A | 0.415 | 0.3488 | 14.7 | 1.26E-04 | 1.325 |
|  | rs4686947 | T/C | 0.2914 | 0.2975 | 0.1403 | 0.708 | 0.9712 |
|  | rs12490232 | A/G | 0.34 | 0.3195 | 1.511 | 0.2189 | 1.097 |
|  | rs12696582 | T/C | 0.2942 | 0.3118 | 1.164 | 0.2807 | 0.9201 |
|  | rs4303859 | C/T | 0.2781 | 0.2687 | 0.3481 | 0.5552 | 1.048 |
|  | rs7631930 | T/C | 0.3446 | 0.3237 | 1.569 | 0.2104 | 1.099 |
|  | rs9870824 | A/T | 0.3414 | 0.3212 | 1.46 | 0.2269 | 1.095 |
|  | rs9852988 | G/A | 0.296 | 0.3139 | 1.198 | 0.2738 | 0.9188 |
|  | rs4377487 | T/G | 0.3634 | 0.367 | 0.04436 | 0.8332 | 0.9846 |
|  | rs4384940 | A/G | 0.3608 | 0.3668 | 0.1225 | 0.7263 | 0.9745 |
|  | rs4686952 | G/A | 0.2769 | 0.2681 | 0.3104 | 0.5774 | 1.045 |
|  | rs12487315 | G/A | 0.3643 | 0.3672 | 0.02937 | 0.8639 | 0.9874 |
