# Supplementary material for: Association Study of Gene LPP in Women with Polycystic Ovary Syndrome
Source: PLoS One. 2012 Oct 3;7(10):e46370. doi: 10.1371/journal.pone.0046370 (PMC3463595; doi:10.1371/journal.pone.0046370)
Supplement: Table S2 — Probes and primers of the three SNPs. F: forward; R: reverse. (DOCX) [file pone.0046370.s003.docx]

**Table S2. Probes and primers of the three SNPs**

| SNPs | Probes | Primers | |
| --- | --- | --- | --- |
| rs715790 | FAM-CTTCTAGGGATTTCCA-MGB | | F-CCTCTAGCTCCCATGATCATTGAC |
|  | VIC-CTTCTAGGGATCTCCA-MGB | | R-GTCAGCTGAGCCTCTCAAAAGC |
| rs4449306 | FAM -TAGCCCATTAAGTTTTCGA-MGB | | F-TTCTACCTTCTGGATGCTTTGATTT |
|  | VIC-TAGCCCATTAAGTTTTAGA-MGB | | R-TGTAGCTTTTAACCATCTCTGTGTCA |
| rs6782041 | FAM-ACTAATACTCGTGTATGCA-MGB | | F-GTTGGTAGGAGAGCAATACCTTTGA |
|  | VIC-ACTAATACTTGTGTATGCAA-MGB | | R-AAAATTACAAACCAATTTCACCAATCT |
